# On the pitfalls of PTV in lung SBRT using type-B dose engine: an analysis of PTV and worst case scenario concepts for treatment plan optimization

**DOI:** 10.1186/s13014-020-01573-9

**Published:** 2020-05-29

**Authors:** Ronnie Wing King Leung, Mark Ka Heng Chan, Chi-Leung Chiang, Matthew Wong, Oliver Blanck

**Affiliations:** 1 Department of Clinical Oncology, TuenMun Hospital, Hong Kong, SAR, China; 2 Department of Radiotherapy, West German Cancer Center, University Hospital Essen, University of Duisburg-Essen, Hufelandstraße 55, 45147 Essen, Germany; 3 Department of Radiotherapy, University Hospital Essen, Kiel Campus, 24105 Kiel, Germany; 4 Department of Clinical Oncology, The University of Hong Kong, Hong Kong, China

## Abstract

**Background:**

PTV concept is presumed to introduce excessive and inconsistent GTV dose in lung stereotactic body radiotherapy (SBRT). That GTV median dose prescription (*D*_*50*_) and robust optimization are viable PTV–free solution (ICRU 91 report) to harmonize the GTV dose was investigated by comparisons with PTV–based SBRT plans.

**Methods:**

Thirteen SBRT plans were optimized for 54 Gy / 3 fractions and prescribed (i) to 95% of the PTV (*D*_*95*_) expanded 5 mm from the ITV on the averaged intensity project (AIP) CT, i.e., PTV_ITV_, (ii) to *D*_*95*_ of PTV derived from the van Herk (VH)‘s margin recipe on the mid–ventilation (MidV)–CT, i.e., PTV_VH_, (iii) to ITV *D*_*98*_ by worst case scenario (WCS) optimization on AIP,i.e., WCS_ITV_ and (iv) to GTV *D*_*98*_ by WCS using all 4DCT images, i.e., WCS_GTV_. These plans were subsequently recalculated on all 4DCT images and deformably summed on the MidV–CT. The dose differences between these plans were compared for the GTV and selected normal organs by the Friedman tests while the variability was compared by the Levene’s tests. The phase–to–phase changes of GTV dose through the respiration were assessed as an indirect measure of the possible increase of photon fluence owing to the type–B dose engine. Finally, all plans were renormalized to GTV *D*_*50*_ and all the dosimetric analyses were repeated to assess the relative influences of the SBRT planning concept and prescription method on the variability of target dose.

**Results:**

By coverage prescriptions (i) to (iv), significantly smaller chest wall volume receiving ≥30 Gy (CW_V30_) and normal lung ≥20 Gy (NL_V20Gy_) were achieved by WCS_ITV_ and WCS_GTV_ compared to PTV_ITV_ and PTV_VH_ (*p* > 0.05). These plans differed significantly in the recalculated and summed GTV *D*_*2*_*, D*_*50*_ and *D*_*98*_ (*p* <  0.05). The inter–patient variability of all GTV dose parameters is however equal between these plans (Levene’s tests; *p* > 0.05). Renormalizing these plans to GTV *D*_*50*_ reduces their differences in GTV *D*_*2*_*,* and *D*_*98*_ to insignificant level (*p* > 0.05) and their inter–patient variability of all GTV dose parameters. None of these plans showed significant differences in GTV *D*_*2*_*, D*_*50*_ and *D*_*98*_ between respiratory phases, nor their inter–phase variability is significant.

**Conclusion:**

Inconsistent GTV dose is not unique to PTV concept but occurs to other PTV–free concept in lung SBRT. GTV *D*_*50*_ renormalization effectively harmonizes the target dose among patients and SBRT concepts of geometric uncertainty management.

## Introduction

Stereotactic body radiotherapy (SBRT) for non–small cell lung carcinomas (NSCLC) is typically delivered in free breathing condition. To limit the negative impact of respiration–induced organ motion and setup errors on its clinical benefits, passive motion management is often pursued, using either the internal target volume (ITV) concept or the mid–ventilation (MidV) concept [1]. Alternatively, passive motion management can also be realized by direct incorporation of the tumor motion into the four–dimensional (4D) optimization framework [2].

Regardless of the motion management techniques and setup uncertainty, dose optimization and prescription are invariably performed with respect to the planning target volume (PTV) to ensure, for instances, 95 and 99% PTV coverage by 100 and 90% of the prescription dose (i.e., PTV *D*_*95*_ = 100% and *D*_*99*_ = 90%). As suggested by Lebredonchel et al. [3], when type**–**B and Monte Carlo (MC) dose algorithms that model lateral electronic equilibrium (LED) are directly used to optimize to PTV *D*_*95*_ a high flux of photon fluence would have to be deposited in the low density lung tissue surrounding the gross tumor volume (GTV). As a consequence, increase of dose in the lung may occur. Worse still, the GTV dose may experience increased variability only during treatment delivery as the tumor moves in and out of the high photon fluence zone over the breathing cycles. As a workaround Lacornerie et al. [4] proposed to use type**–**A algorithm to optimize a homogeneous fluence for which the dose distribution is ultimately calculated and renormalized to the desired prescription level using the more accurate type–B /MC algorithms. In fact, most of the major treatment planning systems (TPS) adapts type**–**A dose engines to increase the speed of inverse optimization for intensity**–**modulated (IMRT) or volumetric modulated**–**arc radiotherapy (VMAT). Type**–**B dose engine is only used at certain intermediate steps as a background dose, the so**–**called intermediate dose, during subsequent optimization to minimize the impact of the dose prediction and optimization convergence errors [5–7].

The latest published International Commission on Radiation Units and Measurements (ICRU) report 91 [8] continues to recommend treatment dose prescription based on PTV coverage (ICRU 91 coverage prescription) while acknowledging the increased variability of the internal GTV dose for lung SBRT using an advanced dose calculation engine. Potential solutions to improve the consistency in the reported dose and hence treatment outcomes were discussed in the report using the GTV median dose *D*_*50*_ prescription and robust optimization (RO) but no further guidelines were provided. Following up the ICRU report 91 recommendations eight ACROP (Advisory Committee on Radiation Oncology) contributing centers have recently reported the variation of their prescription practices, which led to large inter–institutional and for four centers even large intra–institutional variations of the GTV/ITV doses [9]. The ACROP further made five additional clarifications, one of them recommending a minimum GTV biological equivalent mean dose of 150 Gy. Another preliminary study from one ACROP center also demonstrated superior inter–patient variability by prescription/renormalization to ITV *D*_*50*_ to prescriptions by PTV and ITV *D*_*98*_ [10]. However, their results did not concern geometric uncertainty of the GTV. Current studies supporting the GTV median and mean dose optimization and prescription were mostly based on real**–**time tumor tracking SBRT where tumor motion was largely constrained. More importantly, very few clinical outcomes have ever been published [11, 12]. The impact of respiration motion on the variability of target dose is still unknown for the GTV *D*_*50*_ prescription/renormalization methods.

Unlike for proton therapy where RO has been in routine clinical practice [13], the clinical role of RO in photon therapy remains relatively undefined and exploratory. Since RO was introduced to the commercial TPS, there have been a few studies of its clinical application to lung SBRT but mainly focusing on the dosimetric benefits and validating the degree of robustness in reality [14]. For two example patient cases, Zhang et al. [15] showed that combining robust optimization with ITV − based prescription by *D*_*95*_ resulted in indistinguishable dose volume histograms (DHV) of the ITV obtained on multiple breathing instances for a typical tumor motion of 1 cm. In another phantom study, Archibald−Heeren et al. modeled the tumor motion displacement as independent scenarios and performed RO for the worst case scenario (WCS) [16]. They similarly found relatively stable tumor doses for displacement up to 2 cm by optimizing and prescribing to GTV *D*_*99*_. However, the potential of RO to overcome the limitations of PTV has never been explored for the median dose *D*_*50*_ prescription.

In the specific context of respiration**–**induced GTV displacement, the present study aims to validate the hypotheses

1. that using type–B dose engine with the PTV concept for dose optimization and prescription introduces significant variability of target dose and
2. that RO (by the worst case method in this study)–based planning is a viable alternative to the PTV concept in lung SBRT and,
3. that prescription by GTV median dose (*D*_*50*_) can minimize the inter**–**patient and inter**–**technique variability of the reported GTV dose.

For the first argument to be valid, we hypothesized that the GTV received significantly variable doses between breathing phases. Two PTV–based optimization adapting the ITV and MidV concepts were tested using the ICRU 91 coverage prescription method. To validate the second argument, we repeated the assessment of the first argument for two WCS–based robustness optimization (hereafter called WCS optimization).The first approach is identical to Liang et al. [14] that used the ITV concept for motion encompassing. The second approach deployed all 4DCT images as independent breathing scenarios for robustness optimization. Furthermore, the dosimetric robustness was assessed by comparing the relative number of incidences that a certain target and OAR dose limit was violated in different respiratory phases. For argument 1 and 2, the inter–patient variability of GTV dose resulting from the PTV– and WCS–optimized plans were also compared. To test the third argument, all PTV and WCS–optimized plans that were prescribed by coverage according the ICRU 91 recommendation were renormalized to the GTV median dose *D*_*50*_ and the above analyses were repeated.

Findings from this study are expected to provide important insight into the combination of SBRT planning concept and prescription method that produces the optimal dosimetric quality and robustness in target and organ dose during treatment, which will subsequently improve the consistency in dose reporting and multi–center clinical outcome assessment.

## Methods and materials

### Patient selection and pre–treatment preparation

Fourteen consecutive patients with peripherally**–**located lung tumors who previously received SBRT were selected for this retrospective planning study.

Helical four–dimensional computed tomography (4DCT) scan of each patient was acquired in 2 mm axial slices and binned into ten datasets according to respiratory phase. Using all the phase– binned 4DCT datasets an average intensity projection (AIP) image dataset was also generated on the RayStation (RaySearch Laboratories, Stockholm, Sweden; version 8a) treatment planning system (TPS).

### Definition of target and normal organs

The GTV was firstly defined on one of the 4DCT dataset that was closest to the mid–ventilation (MidV) phase (GTV_MidV_) [17]. It was then transformed to all other phases according to the deformation vector fields (DVFs) derived from the anatomically constrained deformation algorithm ANACONDA [18]. Finally, these GTVs from different phases were rigidly transferred onto the AIP images to produce the internal target volume (ITV). The above process also applied to the definition of normal organs.

### Treatment planning strategies to motion encompassing

#### PTV–based optimization

Two margin–based approaches were studied by optimizing to (i) the PTV expanded uniformly by 5 mm from the ITV, denoted as PTV_ITV_, on the AIP images, and (ii) the PTV expanded from the GTV_MidV_ by $2.5{\sum}_{setup}+\beta \sqrt{{\sigma_{setup}}^2+{\sigma_{motion}}^2+{\sigma_p}^2}-{\beta \sigma}_p$ using the van Herk (VH)‘s margin recipe [19], denoted as PTV_VH_, on the MidV CT. *Σ*_*setup*_ and *σ*_*setup*_ are the residual systemic and random errors in the tumor position including patient motion and tumor baseline drift after the online 4D cone beam computed tomography (4DCBCT) setup correction (i.e., intrafractional positioning error), *β* = 0.52 (at a mean prescription isodose line ~ 70%) and *σ*_*p*_ = 6.4, respectively. The motion amplitude of individual tumor is modeled as *σ*_*motion*_ = 1/3 amplitude [19]. The GTV displacement due to respiration was implicitly accounted for by the ITV and the MidV PTV concepts, respectively.

Two partial volumetric–modulated arcs treatment (VMAT) were created using the rayArc optimization algorithm. The rayArc optimization process uses a type–A pencil–beam dose engine. For all VMAT optimization, a type–B collapsed cone convolution–superposition (CCCS) dose engine was introduced at the 15th iteration to calculate intermediate dose as a background dose for subsequent optimization. At the end of the VMAT optimization a final dose was calculated by CCCS. Each time further VMAT optimization was pursued the final dose was taken to have the same effect as an intermediate dose. The final optimized dose was prescribed to PTV *D*_*95*_ at 65–75% isodose line in all cases. A total dose of 54 Gy for three fractions was prescribed in all cases. Dose–volume histogram (DVH) limits to different OARs were referenced from the Radiation Therapy Oncology Group (RTOG) 0236 trial [20] and the German Society of Radiation Oncology (DEGRO) guidelines [21].

#### Robustness optimization

In RayStation, PTV–free planning can be realized by robustness optimization (RO) based on the composite worst case method [22]. The setup uncertainty is discretized into a set of scenarios whose actual number (*n*_*s*_) depends on the size of the error. Together with nominal scenario corresponding to the planning CT with no assumed error, the DVH objectives are optimized for the worst case scenario (WCS) in which a robust function attaints its highest value. It is important to note that RO in RayStation does not treat the systematic and the random errors separately. Following ref. [23], the WCS can be approximated only for the systematic error, with the random error approximated as an additional systematic contribution. Based on the same *Σ*_*setup*_ with *σ*_*setup*_ as in the PTV recipe, the final WCS parameters were 3.4 mm (left-right), 5.3 mm (cranio-caudal) and 5.1 mm (antero-posterior) mm. The remaining organ motion of individual patients was accounted for in two ways:

iii) implicitly by the concept of ITV in a static geometry on the AIP image.

iv) explicitly by the WCS method in a dynamic geometry that was realized by utilizing the 4DCT images of all breathing phases.

In the second WCS approach, each image set of the 4DCT composes one scenario where all the setup scenarios are examined. The total number of scenarios to be considered in the WCS optimization is then *n*_*s*_ · *n*_*i*_, where *n*_*i*_ is the number of 4DCT image sets. At each iteration, minimax optimization was applied to one of all scenarios that attains the highest cost of the robust object function, i.e., the WCS. The resultant optimized plan will be robust against not just setup error but also breathing–induced tumor motion and deformation in all ten 4DCT images, hence completely margin–less. In this study, robustness was imposed to all DVH objectives of the target and the OARs.

The same two partial VMAT arcs as applied in the PTV–based planning were optimized to the ITV and the GTV to achieve 99% prescription dose coverage (*D*_*99*_) in the first and the second WCS approaches, denoted as (iii) WCS_ITV_ and (iv) WCS_GTV_, respectively. The same VMAT optimization process as in the PTV–optimization was adapted regarding the dose engines for optimization and prescription.

### Comparative analysis of PTV and WCS optimizations in static geometry

Firstly we assessed the naïve plan optimized according to (i) to (iv) without explicit simulation of the geometric tumor displacement due to respiration. The MidV–CT was used as the common frame where all the dosimetric metrics, including mean, near–minimum and near–maximum dose in the GTV (GTV *D*_*50*_, *D*_*98*_ and *D*_*2*_), relative volume of chest wall (CW) receiving 30 Gy (CW_V30_), relative volume of normal lung (NL) receiving 20 Gy (NL_V20_) and 5 Gy (NL_V5_) and the mean normal lung dose (MLD) were obtained. For this, all PTV_ITV_ and WCS_ITV_ plans that based their optimizations on the AIP images were recalculated on the MidV–CT.

Doses to GTV and OARs of the PTV and WCS optimized plans were compared for their difference by Friedman tests and their variance by Levene’s tests using the Matlab statistics toolbox v.2019b (Mathwork Inc. MA, USA). In cases where the Friedman’s tests return statistical significant at *p–*value < 0.05, *post–hoc* multiple comparison tests were performed with adjusted *p–*values by Bonferroni’s correction.

### Dosimetric variation / robustness of PTV and WCS optimizations in breathing geometry

#### Analysis by individual respiratory phases

If type–B dose engine does induce excessive fluence in the low density PTV border one would expect the dose received by the GTV to be higher in other breathing phases than in the planning phase. As validation, all PTV and WCS–optimized plans were firstly recalculated on every image set of the 4DCT. The resulting doses to the GTV and organs–at–risk (OARs) in individual breathing phases were statistically compared for their difference by Friedman tests and their variance by Levene’s tests separately for the PTV and WCS–optimized plans. The plan robustness was defined in this context by the relative count of instances where the doses to the GTV and OARs deviate from their respective tolerance limits.

#### Analysis over all respiratory phases

Following the line of argument, if the PTV concept using type–B dose engine introduces excessive photon fluence the GTV would eventually accumulate significant higher dose from multiple displaced positions in the respiration cycle. As the ultimate validation, the calculated doses in individual 4DCT phase images were summed deformably according to DVFs back onto the reference MidV–CT for every plan. Such deformably accumulated dose is unequivocally referred to as summed dose throughout the text. Similar to the evaluation in the static geometry, the summed doses to GTV and OARs were compared among all PTV and WCS–optimized plans on the reference MidV–CT for their differences by Friedman tests and their variances by Levene’s tests.

The overall plan robustness was defined in this context as the dosimetric changes due to the motion effect from static to dynamic geometry and was assessed separately for different PTV and WCS WCS–optimized plans by the Wilcoxon’s signed–ranked test.

### Dosimetric implication of prescription by GTV median dose *D*_*50*_

According to ICRU 91 report and other follow–up studies [1, 3, 16], GTV *D*_*50*_ prescription was further explored for its potential in mitigating the variability of GTV dose under the circumstances of GTV displacement by respiration. For this, all final PTV and WCS–optimized plans were renormalized so that GTV *D*_*50*_ equals 54 Gy on respective primary planning CT images. Dosimetric and statistical analyses were then repeated as described above.

## Results

### PTV– and WCS–based SBRT using ICRU 91 recommended coverage prescription

#### Dosimetric analysis in static geometry

**Fig. 1:**
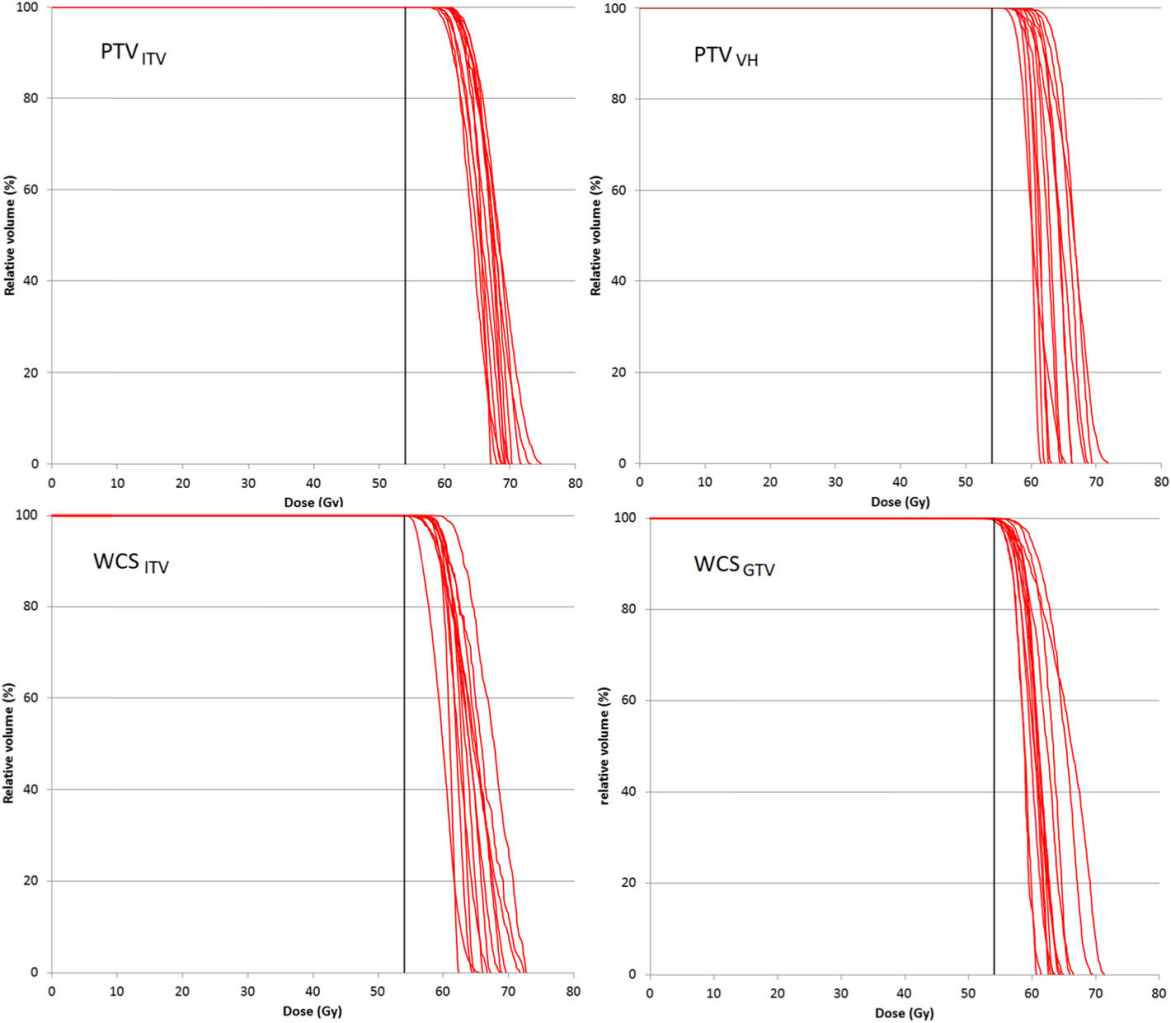
Each red line represents the GTV dose volume histogram of individual patient resulting from PTV– and WCS–based SBRT plans. The black vertical line indicates the prescription dose at 54 Gy

In the condition where no tumor displacement is concerned, all PTV and WCS–optimized plans achieved the dose constraints following the RTOG 0236 and guidelines on the reference mid–ventilation images, except for CW. In general, WCS–optimized plans produced lower doses than PTV–optimized plans not just in the OARS but also in the GTV, as summarized in Table 1. Figure 1 shows the DVH of the GTV obtained on the MidV–CT for individual patients.

**Table 1:** Means and standard deviations of dose volume histogram (DVH) metrics in target and normal organs over 13 patients. All plans were prescribed by coverage according to ICRU 91

	PTV_ITV_^(i)^	PTV_VH_^(ii)^	WCSC_ITV_^(iii)^	WCS_GTV_^(iv)^	*p*-value< 0.05
GTV					
*D*_*98*_ (Gy)	60.6 ± 1.8 (56.1–63.7)	59.6 ± 1.5 (57.0–62.3)	57.8 ± 1.7 (54.8–59.6)	56.4 ± 1.0 (55.2–58.6)	(i)-(iii); (i)-(iv); (ii)-(iv)
*D*_*50*_ (Gy)	65.7 ± 2.2 (59.7–68.1)	63.1 ± 2.2 (60.0–66.5)	63.7 ± 2.2 (59.9–67.2)	60.5 ± 2.1 (56.4–64.8)	(i)-(iv)
*D*_*2*_ (Gy)	69.3 ± 2.3 (65.1–76.3)	65.4 ± 3.0 (61.3–70.7)	67.2 ± 3.5 61.6–72.5)	64.3 ± 2.9 (60.5–70.7)	(i)-(iv)
Chest Wall					
*V*_*30Gy*_ (cm^3^)	26.4 ± 16.5 (4.0–60.9)	19.3 ± 9.9 (1.0–38.1)	14.1 ± 8.7 (0.4–31.4)	12.4 ± 7.7 (0.0–29.5)	(i)-(iii); (i)-(iv); (ii)-(iii); (ii)-(iv)
Lung					
*V*_*5Gy*_ (cm^3^)	18.1 ± 5.7 (3.3–14.0)	18.7 ± 5.0 (3.6–13.5)	18.1 ± 5.8 (3.1–13.4)	17.0 ± 5.6 (2.8–13.4)	(i)-(iv); (ii)-(iv)
*V*_*20Gy*_ (cm^3^)	8.4 ± 3.7 (9.1–31.3)	7.8 ± 3.3 (9.5–28.3)	7.4 ± 3.2 (8.3–30.7)	6.9 ± 3.3 (7.8–29.8)	(i)-(iii); (i)-(iv); (ii)-(iii); (ii)-(iv)
*MLD* (Gy)	4.8 ± 1.7 (2.2–7.9)	4.7 ± 1.5 (2.3–7.2)	4.6 ± 1.6 (2.1–7.1)	4.2 ± 1.5 (1.9–7.1)	(i)-(iv); (ii)-(iv)

Abbreviation: *GTV* gross tumor volume, *D*_*x*_*(Gy)* Dose to x % of the GTV, where x = 98, 50 and 2 represent the near–minimum, median and near–maximum dose, respectively, *V*_*xGy*_*(cm*^*3*^*)* volume receiving at least x Gy, *MLD (Gy)* mean normal lung dose; *post*–*hoc* pairwise comparisons showing *p*-values < 0.05 are indicated when the Freidman’s tests were found statistical significant

On individual patient basis, CW_V30_ was not met in 3 cases by PTV_ITV_ and 1 case by PTV_VH_ and WCS_ITV_ while it was met in all cases by WCS_GTV_. For NL_V20_, PTV_ITV_ resulted in 6 minor deviations (within 10–15%) and PTV_VH_ showed 5 minor deviations according to the RTOG 0236 dose constraint. By contrast, there were 4 and 2 minor deviations resulted from WCS_ITV_ and WCS_GTV_ plans, respectively. The separation between PTV– and WCS–based SBRT plans is more pronounced in NL_V20_ and between PTV_ITV_, PTV_VH_ and WCS_GTV_. We found that WCS_GTV_ is able to reduce NL_V5_, on average, by 6.1%, NL_V20_ by 17.9% and MLD by 12.5% comparing to PTV_ITV_.

Figure 1 shows the inter–patient variability of the GTV doses. Variances of each dose metrics between all PTV–based and WCS–based SBRT plans were statistically tested (Levene’s tests) and were found significant for neither the GTV (*D*_*98*_, *D*_*50*_ and *D*_*2*_; all *p* > 0.05) nor the OARs (CW_V30_, NL_V5_ and NL_V20_).

#### Dosimetric analysis by individual breathing phases

Recalculating the PTV– and WCS–optimized plans on every image set of the 4DCT found GTV *D*_*50*_ ≥ 54Gy in all cases. PTV_ITV_ produced *D*_*98*_ > 54 Gy in all patients. There are one PTV_VH_ plan in one phase, two WCS_ITV_ plans in one phase and one WCS_GTV_ plan in three phases showing *D*_*98*_ < 54 Gy towards principally the end inhalation. The maximum differences of *D*_*98*_ (± 1 standard deviation; SD) between all 4DCT phases are 5.7% ± 1.3, 11.4% ± 3.0, 6.5% ± 1.6 and 6.1% ± 1.5% for PTV_ITV_, PTV_VH_, WCS_ITV_, and WCS_GTV_, and for GTV *D*_*50*_ 1.6% ± 0.5, 3.4% ± 0.8, 1.5% ± 0.4 and 2.1% ± 0.5%, respectively.

**Fig. 2:**
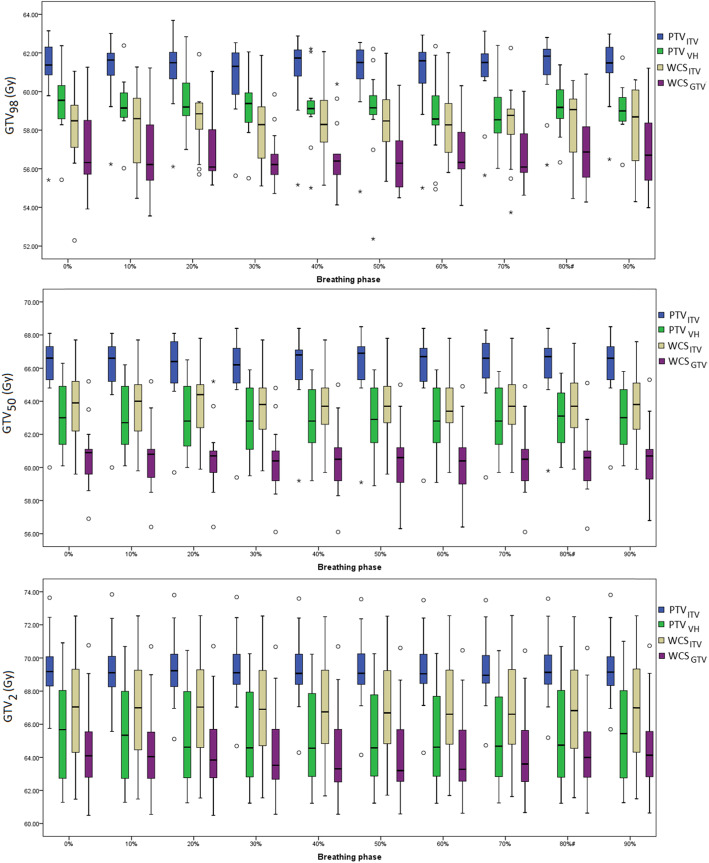
Box–plots show the phase–to–phase variations of GTV *D*_*98*_, *D*_*50*_ and *D*_*2*_ calculated over 13 patients for PTV and WCS–based SBRT plans. Line across the box indicates the median and the box indicate the inter–quartile range (IQR). The whiskers indicate the highest and lowest values which are no greater than 1.5 times the IQR. The outliners (opened circles) and the extremes (asterisks) are cases with values between 1.5 and 3 times, and more than 3 times the IQR, respectively

Figure 2 shows the variations of GTV *D*_*98*_, *D*_*50*_ and *D*_*2*_ for the 13 cases across ten breathing phases. Over all patients, none of the PTV and WCS–optimized plans showed statistical importance in their differences for GTV *D*_*98*_ and *D*_*50*_ (*p* > 0.05) between respiratory phases and significance was found only for *D*_*2*_ with PTV_VH_ (Table 2). Furthermore, none of the SBRT planning concepts shows significant inter–phase variability (in terms of their variances) in GTV *D*_*98*_, *D*_*50*_ and *D*_*2*_ (*p* > 0.05).

**Table 2:** Ranges of the mean and one standard deviation (SD) of the dose volume histogram metrics over10 breathing phases in13 patients. All plans were prescribed by coverage according to ICRU 91

	PTV_ITV_	PTV_VH_		WCS_ITV_		WCS_GTV_		
	Range		Range		Range		Range	
	Mean (*p*-value)	SD (*p*-value)	Mean (*p*-value)	SD (*p*-value)	Mean (*p*-value)	SD (*p*-value)	Mean (*p*-value)	SD (*p*-value)
GTV								
*D*_*98*_ (Gy)	55.7–62.5 (0.65)	0.3–1.0 (1.00)	55.5–62.2 (0.32)	0.2–1.8 (0.91)	55.0–61.5 (0.96)	0.3–1.1 (0.99)	54.8–60.5 (0.90)	0.2–1.2 (0.70)
*D*_*50*_ (Gy)	59.6–68.3 (0.38)	0.1–0.3 (1.00)	60.0–65.8 (0.58)	0.1–0.8 (1.00)	59.7–67.6 (0.82)	0.1–0.3 (1.00)	56.4–65.1 (0.84)	0.1–0.5 (1.00)
*D*_*2*_ (Gy)	64.9–73.6 (0.32)	0.0–0.6 (1.00)	61.2–70.5 (< 0.05)	0.0–0.7 (1.00)	61.6–72.5 (0.42)	0.0–0.3 (1.00)	60.6–70.6 (0.43)	0.0–0.5 (1.00)
Chest Wall								
*V*_*30Gy*_ (cm^3^)	9.3–73.1 (< 0.01)	0.7–5.3 (1.00)	2.8–39.9 (< 0.01)	0.8–4.8 (1.00)	1.6–31.6 (< 0.01)	0.3–4.4 (1.00)	1.8–29.9 (< 0.01)	0.3–3.8 (1.00)
Lung								
*V*_*5Gy*_ (cm^3^)	9.4–29.0 (0.06)	0.1–1.7 (0.91)	9.8–25.9 (< 0.05)	0.1–1.9 (1.00)	8.6–27.8 (< 0.05)	0.1–2.0 (1.00)	8.0–27.0 (0.09)	0.1–2.0 (1.00)
*V*_*20Gy*_ (cm^3^)	3.6–15.1 (0.16)	0.1–3.7 (1.00)	3.8–14.8 (0.09)	0.1–1.3 (1.00)	3.3–14.8 (0.07)	0.1–1.2 (1.00)	3.0–14.0 (0.15)	0.1–1.3 (1.00)
*MLD* (Gy)	2.3–8.3 (< 0.01)	0.0–0.6 (1.00)	2.4–7.9 (< 0.01)	0.0–0.5 (1.00)	2.2–7.8 (< 0.01)	0.0–0.5 (1.00)	2.0–7.4 (< 0.01)	0.0–0.5 (1.00)

Note: *p* values were obtainedfrom Freidman’s tests and Levene’s tests comparing the differences and the variances between ten breathing phases, respectively, in 13 patients per SBRT optimization method

Abbreviations are the same as in Table 1

#### Dosimetric analysis over all breathing phases

**Fig. 3:**
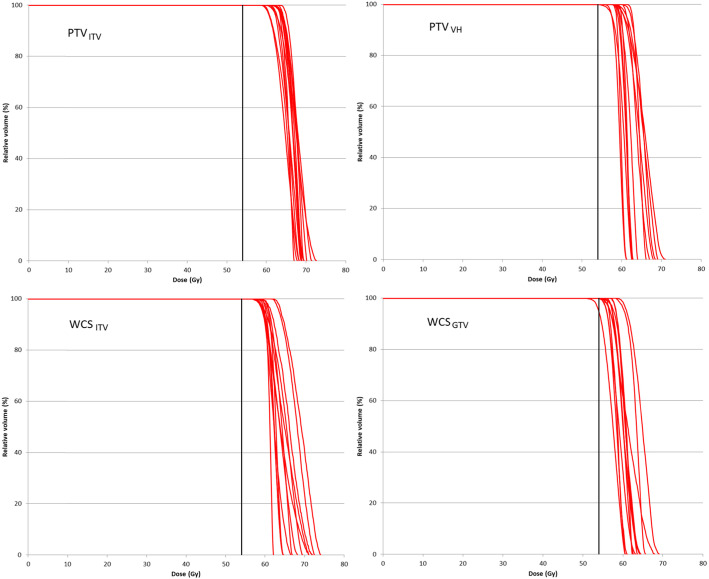
Each red line represents the GTV DVH of individual patient obtained from the accumulated dose for PTV and WCS–based SBRT plans. The black vertical line indicates the prescription dose at 54 Gy

Table 3 shows the results of the accumulated doses obtained from the PTV and WCS– optimized plans. The accumulated GTV *D*_*98*_ achieved 54 Gy in all plans. The changes of GTV *D*_*98*_, *D*_*50*_ and *D*_*2*_ are, on average, largely limited to 1.0 Gy. Figure 3 shows the DVH of the GTV obtained from the accumulated dose for individual patients. The inter–patient variability of doses to both the GTV and the OARs were tested to be equal among all plans (Levene’s tests; all *p* > 0.05).

**Table 3:** Accumulated dose volume histograms (DVH) over the entire breathing cycle for PTV and WCS optimized plans based on prescription by coverage according to ICRU 91

		PTV_ITV_^(i)^	PTV_VH_^(ii)^	WCSC_ITV_^(iii)^	WCS_GTV_^(iv)^	
	Motion effect (Wilcoxon’s)	Mean ± SD (range)	Mean ± SD (range)	Mean ± SD (range)	Mean ± SD (range)	*(i)* vs.*(ii)* vs.*(iii)* vs.*(iv) p-value* < 0.05
GTV						
*D*_*98*_ (Gy)		61.5 ± 1.8 (56.3–63.0)	59.7 ± 1.6 (56.5–62.5)	59.0 ± 1.8 (55.5–62.0)	57.2 ± 1.9 (55.1–61.3)	(i)-(iii); (i)-(iv); (ii)-(iv)
	*p-value*	0.003	1.000	0.022	0.092	
*D*_*50*_ (Gy)		65.7 ± 2.2 (59.6–67.9)	62.8 ± 2.1 (59.8–65.6	63.2 ± 2.2 (59.7–67.2)	60.3 ± 2.2 (56.1–65.0)	(i)-(iv)
	*p-value*	0.581	0.092	0.022	0.267	
*D*_*2*_ (Gy)		69.1 ± 2.3 (64.5–73.3)	65.2 ± 3.0 (61.1–70.1)	66.9 ± 3.6 (61.5–72.4)	64.0 ± 2.8 (60.5–70.3)	(i)-(iv)
	*p-value*	< 0.01	< 0.01	< 0.01	< 0.01	
Chest Wall						
*V*_*30Gy*_ (cm^3^)		28.5 ± 18.7 (6.3–72.4)	20.0 ± 10.6 (1.1–41.6)	16.9 ± 9.0 (1.7–31.4)	13.0 ± 8.4 (1.0–32.5)	(i)-(iii); (i)-(iv); (ii)-(iv); (iii)-(iv);
	*p-value*	0.20	0.02	< 0.01	< 0.01	
Lung						
*V*_*5Gy*_ (cm^3^)		16.5 ± 5.8 (7.7–30.0)	17.3 ± 5.1 (8.0–26.7)	16.8 ± 5.7 (7.5–28.7)	16.1 ± 5.6 (7.2–28.3)	(ii)-(iv)
	*p-value*	0.14	0.17	0.20	0.03	
*V*_*20Gy*_ (cm^3^)		8.3 ± 3.8 (3.6–15.4)	7.8 ± 3.3 (3.6–13.8)	7.7 ± 3.7 (3.3–15.1)	7.0 ± 3.4 (2.8–13.4)	(i)-(iii); (i)-(iv); (ii)-(iv); (iii)-(iv)
	*p-value*	0.27	0.11	0.25	0.10	
*MLD* (Gy)		4.0 ± 1.6 (1.8–7.2)	4.1 ± 1.3 (1.9–6.1)	3.9 ± 1.4 (1.8–6.3)	3.8 ± 1.4 (1.7–6.3)	(i)-(iv); (ii)-(iv)
	*p-value*	0.06	0.38	0.22	0.18	

Abbreviations are the same as in Table 1

Note: The motion effect is the evaluated by comparing the recalculated and accumulated dose on the MidV–CT

For CW_V30_, one more patient failed the CW_V30_ tolerance (i.e., total 4 patients) in PTV_ITV_ while one patient failed in PTV_VH_, WCS_ITV_ and WCS_GTV_. For NL_V20_, one minor deviation became major deviations in PTV_ITV_, one less minor deviations (total 4 cases) in PTV_VH_ and the same number of minor deviations in the WCS group were found after dose summation over the tumor’s excursion along the breathing cycle.

### PTV– and WCS–based SBRT by GTV median dose renormalization

#### Dosimetric analysis in static geometry

**Fig. 4:**
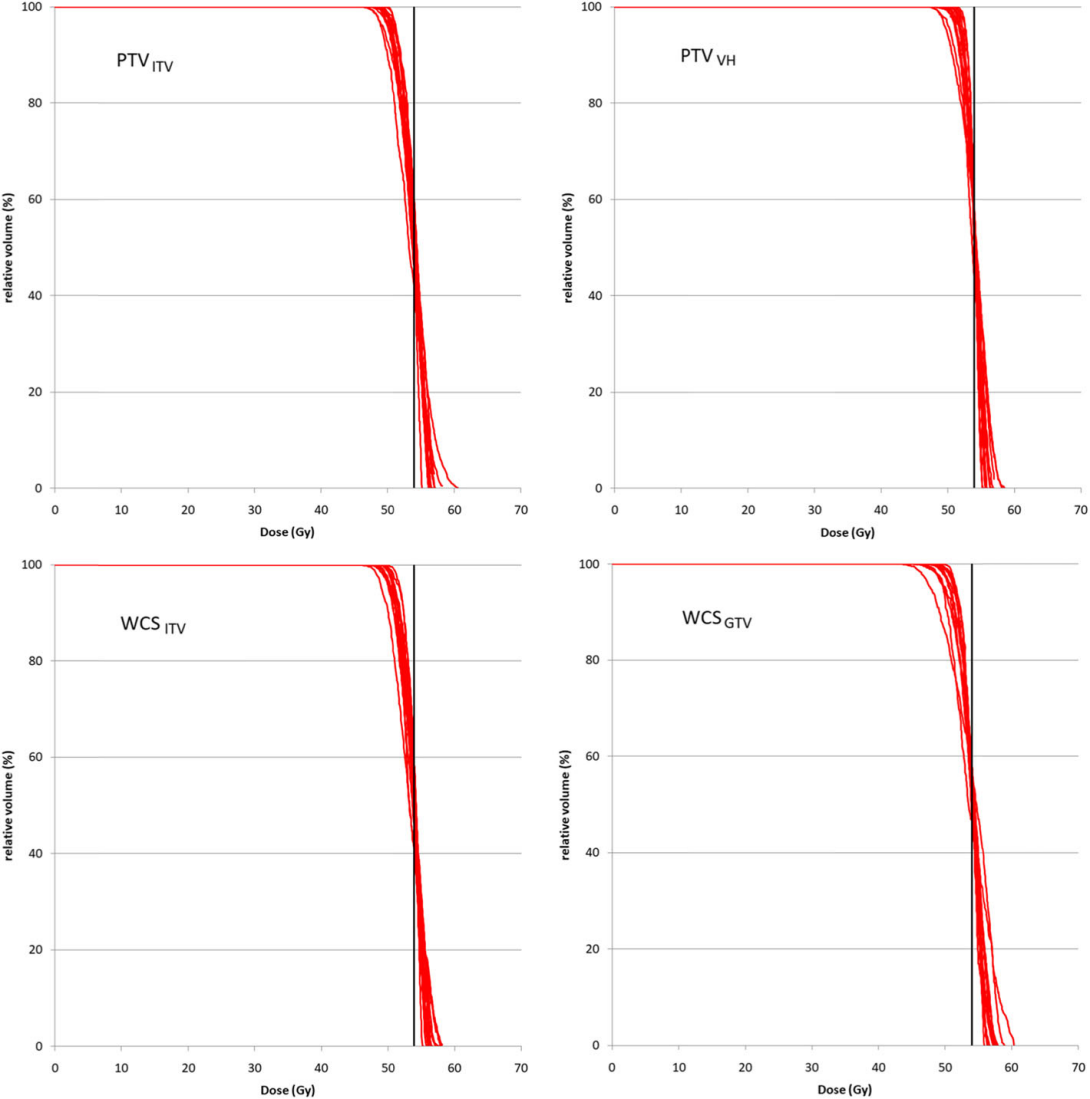
Each red line represents the GTV DVH of individual patient obtained from PTV and WCS–based SBRT plans by GTV *D*_*50*_ prescription. The black vertical line indicates the prescription dose at 54 Gy

Figure 4 shows the DVH family of the GTV after renormalized to the GTV *D*_*50*_. Table 4 shows the resulting doses after renormalization to GTV *D*_*50*_. Insignificant differences were found for all dose metrics, except GTV *D*_*50*_ and CW_V30_ (*p* <  0.05) among PTV and WCS–optimized plans. Furthermore, equal variances were found for all GTV dose metrics between optimization scenarios, except for GTV *D*_*50*_.

**Table 4:** Means and standard deviations of dose volume histogram (DVH) metrics in target and normal organs over 13 patients. All plans were renormalized based on GTV median dose *D*_*50*_

	PTV_ITV_^(i)^	PTV_VH_^(ii)^	WCSC_ITV_^(iii)^	WCS_GTV_^(iv)^	*p*-value < 0.05
GTV					
*D*_*98*_ (Gy)	50.3 ± 0.6 (48.7–51.0)	51.3 ± 1.0 (49.1–52.4)	50.0 ± 1.4 (47.9–52.5)	49.8 ± 1.2 (46.4–51)	(ii)-(iv)
*D*_*50*_ (Gy)	54.1 ± 0.2 (53.8–54.5)	54.0 ± 0.0 (54.0–54.0)	54.1 ± 0.3 (53.5–54.6)	54.0 ± 0.0 (54.0–54.0)	
*D*_*2*_ (Gy)	56.7 ± 0.9 (55.1–58.8)	56.0 ± 0.9 (55.1–57.7)	57.0 ± 1.1 (55.0–58.9)	56.8 ± 0.7 (55.8–58.4)	(ii)-(iii)
Chest Wall					
*V*_*30Gy*_ (cm^3^)	8.2 ± 7.9 (0.0–24.5)	8.1 ± 6.4 (0.0–18.8)	6.7 ± 4.8 (0.0–15.0)	6.6 ± 4.8 (0.0–15.4)	
Lung					
*V*_*5Gy*_ (cm^3^)	17.1 ± 5.3 (2.6–13.6)	17.9 ± 4.3 (2.9–11.7)	17.4 ± 5.1 (2.6–11.9)	16.7 ± 5.1 (2.4–12.3)	(ii)-(iv)
*V*_*20Gy*_ (cm^3^)	6.6 ± 3.3 (7.7–30.0)	6.7 ± 3.0 (8.0–26.8)	6.2 ± 3.0 (7.4–28.7)	6.1 ± 3.1 (7.1–28.4)	(ii)-(iii); (ii)-(iv)
*MLD* (Gy)	3.9 ± 1.6 (1.8–7.2)	4.0 ± 1.3 (1.9–6.1)	3.9 ± 1.4 (1.8–6.3)	3.7 ± 1.3 (1.7–6.4)	(ii)-(iv)

Abbreviations are the same as in Table 1

#### Dosimetric analysis by individual breathing phases

**Fig. 5:**
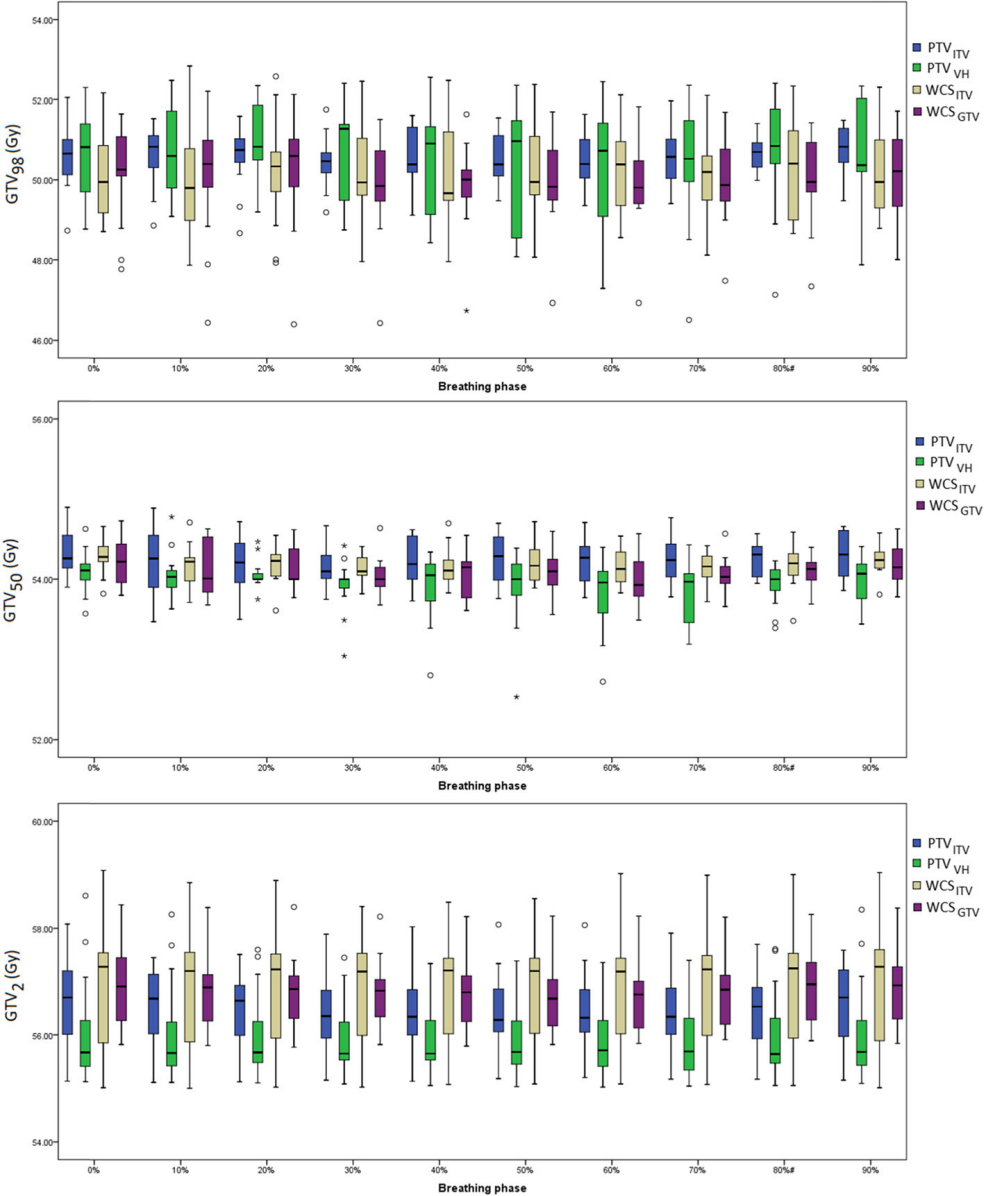
Box–plots show the phase–to–phase variations of GTV *D*_*98*_, *D*_*50*_ and *D*_*2*_ calculated over 13 patients for PTV and WCS–optimized plans based on GTV *D*_*50*_ prescription. Values of the box, whiskers, outliners and extremes are described in Fig. 2; The same 2 Gy scale is set as in Fig. 2

Figure 5 shows the phase–to–phase variations of GTV *D*_*98*_, *D*_*50*_ and *D*_*2*_ after renormalizing the summed GTV *D*_*50*_. Concerning the near–minimum dose *D*_*98*_, the maximum difference (± 1 SD) between all 4DCT phases amounts to 3.3% ± 0.7, 6.6% ± 2.0%, .4.1% ± 0.8 and 5.1% ± 1.2% for PTV_ITV_, PTV_VH_, WCS_ITV_, and WCS_GTV_, and for GTV *D*_*50*_ 2.2% ± 0.6%, 3.4 ± 0.9%, 1.5 ± 0.4, 2.1% ± 0.5%, respectively.

The statistical results of the dose differences for GTV and selected OARs between phases are given in Table 5. None of the PTV and WCS–based SBRT concepts showed statistical significance in the difference of GTV dose between phases, except for *D*_*2*_ of the renormalized PTV_ITV_ plans. Furthermore, the inter–phase variability of GTV *D*_*98*_, *D*_*50*_ and *D*_*2*_ was tested to be equal for all PTV and WCS–optimized plans.

**Table 5:** Ranges of the mean and one standard deviation (SD) of the dose volume histogram metrics over10 breathing phases in13 patients. All plans were normalized based on GTV median dose *D*_*50*_

	PTV_ITV_		PTV_VH_		WCS_ITV_		WCS_GTV_	
	Range		Range		Range		Range	
	Mean (*p*-value)	SD (*p*-value)	Mean (*p*-value)	SD (*p*-value)	Mean (*p*-value)	SD (*p*-value)	Mean (*p*-value)	SD (*p*-value)
GTV								
*D*_*98*_ (Gy)	49.2–51.5 (0.82)	0.2–0.5 (0.91)	48.2–52.4 (0.32)	0.1–0.9 (0.67)	48.4–52.3 (0.90)	0.2–0.6 (0.99)	51.4–47.1 (0.59)	0.1–1.0 (0.98)
*D*_*50*_ (Gy)	54.0–54.6 (0.57)	0.1–0.4 (0.40)	53.5–54.4 (0.24)	0.0–0.7 (0.25)	54.0–54.6 (0.29)	0.0–0.2 (0.86)	53.7–54.5 (0.76)	0.0–0.4 (0.21)
*D*_*2*_ (Gy)	55.2–57.8 (< 0.05)	0.0–0.6 (1.00)	55.1–57.6 (0.25)	0.0–0.6 (1.00)	55.0–58.8 (0.49)	0.0–0.3 (1.00)	55.8–58.3 (0.10)	0.0–0.4 (1.00)
Chest Wall								
*V*_*30Gy*_ (cm^3^)	0.1–32.8 (< 0.01)	0.0–4.4 (0.99)	0.1–19.1 (< 0.01)	0.0–3.0 (0.97)	0.1–14.9 (< 0.01)	0.0–2.6 (0.86)	0.1–16.4 (< 0.01)	0.0–2.5 (0.99)
Lung								
*V*_*5Gy*_ (cm^3^)	7.9–28.0 (0.08)	0.1–1.6 (1.00)	8.2–24.5 (< 0.05)	0.1–1.9 (1.00)	7.7–26.6 (< 0.05)	0.1–1.6 (1.00)	7.4–25.7 (< 0.03)	0.1–2.0 (1.00)
*V*_*20Gy*_ (cm^3^)	2.8–12.8 (0.09)	0.1–1.4 (1.00)	3.1–12.9 (0.11)	0.1–1.2 (1.00)	2.7–12.2 (0.08)	0.1–1.3 (1.00)	2.6–12.0 (< 0.05)	0.0–1.3 (1.00)
*MLD* (Gy)	1.8–6.9 (< 0.01)	0.0–0.5 (1.00)	2.0–6.5 (< 0.01)	0.0–0.5 (1.00)	1.9–6.5 (< 0.01)	0.0–0.5 (1.00)	1.8–6.1 (< 0.01)	0.0–0.5 (1.00)

Note: *p* values were obtainedfrom Freidman’s tests and Levene’s tests comparing the differences and the variances between ten breathing phases, respectively, in 13 patients per SBRT planning method

Abbreviations are the same as in Table 1

#### Dosimetric analysis over all breathing phases

**Fig. 6:**
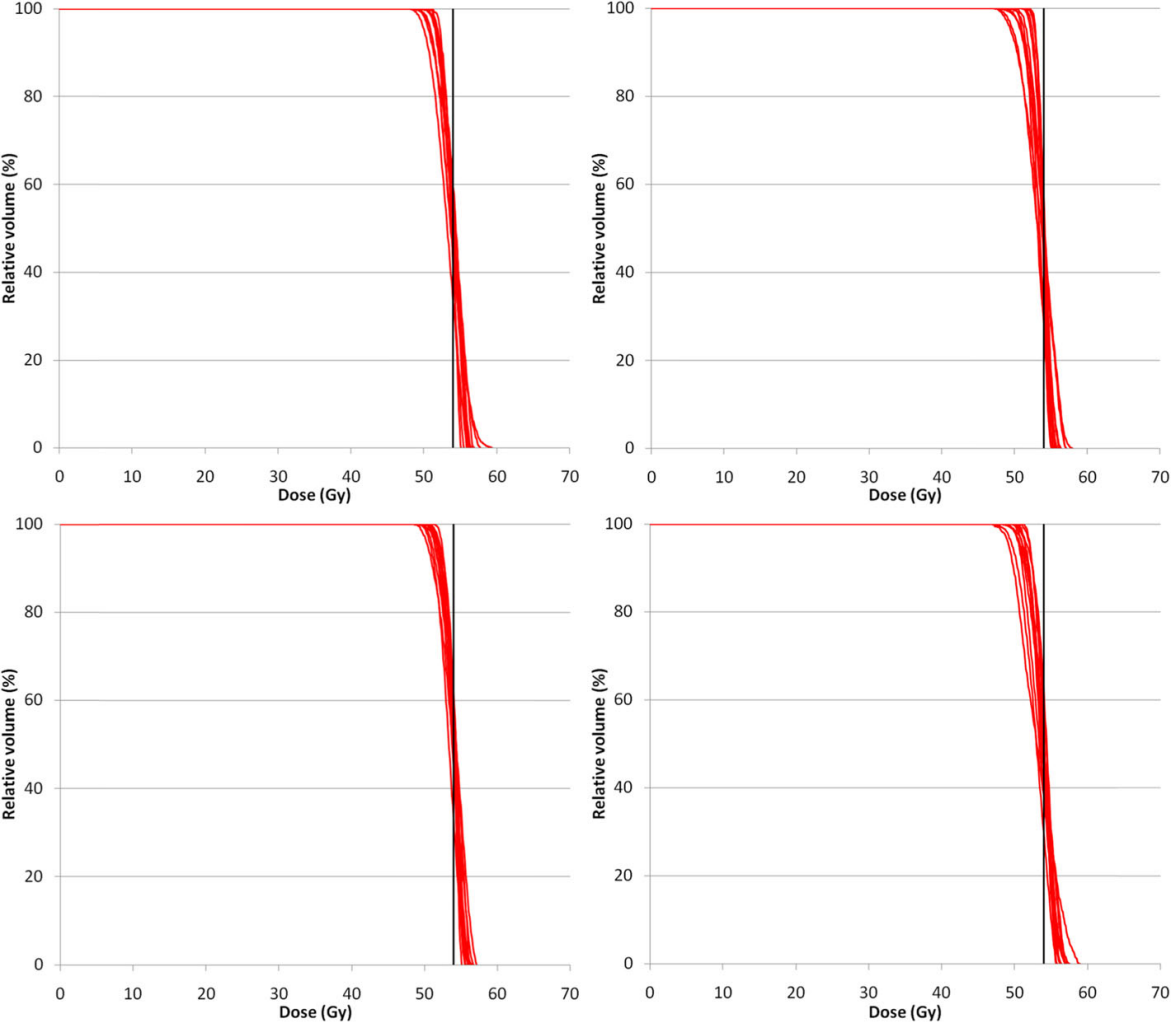
Each red line represents the GTV DVH of individual patient obtained from the accumulated dose after prescribing to GTV *D*_*50*_. The black vertical line indicates the prescription dose at 54 Gy

After dose accumulation, the GTV *D*_*98*_, *D*_*50*_ and *D*_*2*_ and MLD change by less than 0.5 Gy, on average. Figure 6 shows the resulting DVH of the GTV obtained for individual patients. The absolute changes of CW_V30_ and NL_V5_ by 0.6 cm^3^ are considered negligible despite statistical significances. Detailed results for the GTV *D*_*50*_ renormalized plans are given in Table 6. Furthermore, variances of all GTV dose metrics were tested to be equal among all renormalized PTV– and WCS–optimized plans.

**Table 6:** Accumulated dose volume histograms (DVH) over the entire breathing cycle. All plans were renormalized to GTV median dose *D*_*50*_

		PTV_ITV_^(i)^	PTV_VH_^(ii)^	WCSC_ITV_^(iii)^	WCS_GTV_^(iv)^	
	motion effect (Wilcoxon’s)	Mean ± SD (range)	Mean ± SD (range)	Mean ± SD (range)	Mean ± SD (range)	*(i)* vs.*(ii)* vs.*(iii)* vs.*(iv) p-value* < 0.05
GTV						
*D*_*98*_ (Gy)		50.8 ± 0.6 (49.6–51.7)	51.0 ± 1.3 (48.5–52.6)	50.4 ± 1.3 (48.6–52.5)	50.3 ± 1.1 (47.4–51.5)	
	*p-value*	0.003	1.000	0.003	0.92	
*D*_*50*_ (Gy)		54.0 ± 0.2 (53.7–54.4)	53.8 ± 0.3 (53.1–54.2)	54.0 ± 0.2 (53.7–54.3)	53.9 ± 0.2 (53.4–54.3)	(ii)-(iv)
	*p-value*	0.267	0.022	0.092	0.092	
*D*_*2*_ (Gy)		56.4 ± 0.7 (55.0–57.7)	55.8 ± 0.8 (55.0–57.4)	56.7 ± 1.0 (54.9–58.7)	56.5 ± 0.7 (55.6–58.0)	
	*p-value*	< 0.01	< 0.01	< 0.01	< 0.01	
Chest Wall						
*V*_*30Gy*_ (cm^3^)		8.8 ± 8.4 (0.0–27.2)	8.7 ± 6.9 (0.0–21.2)	7.3 ± 5.5 (0.0–17.5)	7.1 ± 5.3 (0.0–17.5)	
	*p-value*	0.01	0.02	0.02	0.02	
Lung						
*V*_*5Gy*_ (cm^3^)		16.5 ± 5.8 (7.7–30.0)	17.3 ± 5.1 (8.0–26.7)	16.8 ± 5.7 (7.5–28.7)	16.1 ± 5.6 (7.2–28.3)	(ii)-(iv)
	*p-value*	0.01	0.04	0.02	0.03	
*V*_*20Gy*_ (cm^3^)		6.7 ± 3.3 (2.7–13.5)	6.8 ± 3.1 (2.9–12.0)	6.3 ± 3.0 (2.6–11.9)	6.2 ± 3.1 (2.5–12.2)	(ii)-(iii); (ii)-(iv)
	*p-value*	< 0.01	< 0.05	0.02	< 0.01	
*MLD* (Gy)		4.0 ± 1.6 (1.8–7.2)	4.1 ± 1.3 (1.9–6.1)	3.9 ± 1.4 (1.8–6.3)	3.8 ± 1.4 (1.7–6.3)	(ii)-(iv)
	*p-value*	0.01	0.01	< 0.01	< 0.01	

Abbreviations are the same as in Table 1

Note: The motion effect is the evaluated by comparing the recalculated and accumulated dose on the MidV–CT

## Discussion

The criticism on the PTV concept in lung SBRT arises from the notion that its combination with type–B and Monte Carlo (MC) dose optimization would result in excessive and inconsistent GTV dose owing to an artificial increase of photon fluence in the low density lung tissue. Such limitation of current SBRT practice is also recognized in the recent ICRU report 91on prescribing, recording, and reporting of stereotactic treatments with small photon beams. This report further suggested that robust optimization and GTV–based prescriptions are viable options to possibly improve the overall dosimetric accuracy and quality while reducing the variability in dose reporting and potentially even in dose prescription.

By analyzing the dosimetric variability and robustness resulting from two common PTV–based and two other worse case–based robust optimization methods, this study is now able to provide more clarifications to the pitfalls of PTV concept in lung SBRT. Additionally, by analyzing further the dosimetric results by different dose prescription methods according to the ICRU recommended coverage prescription and GTV median dose prescription, we identified the dominant factor that contributes to the variability of GTV dose.

### PTV– and WCS–based SBRT using ICRU 91 recommended coverage prescription

SBRT plans optimized and prescribed to the PTV resulted in significant overexposure to the GTV compared to those plans optimized for WCS as expected. The GTV receives much higher dose, with the GTV median dose *D*_*50*_ about 17 and 22% over the prescription dose for ITV–based and mid–ventilation based PTV optimizations, respectively. Although higher dose to the GTV is generally not a concern and even desired for SBRT, part of the excessive dose is in effect burdened by the surrounding normal organs including the normal lung and chest wall that are encompassed in the PTV. For lesions that are close to the chest wall, the volume receiving ≥30 Gy (V_30_) was reduced significantly by up to 29.5 cm^3^ (74%) and 31.4 (73%) by using WCS optimization on the averaged intensity projection (AIP) image to the ITV and on all 4DCT images directly to the GTV in comparisons to the conventional PTV approach based on the ITV. The dosimetric benefit of WCS optimization to limit the chest wall dose was also reported by Zhang et al. [21]. In their study, 8 of 20 patient plans optimized and prescribed to PTV showed chest wall dose above the limit whereas all WCS plans optimized to the ITV fulfilled the dose constraint. In this study, we showed that WCS optimized to the GTV can further improve the chest wall dose.

Besides the dosimetric inferiority to WCS optimization, the other major pitfall of PTV concept for plan optimization is that inconsistent GTV doses between individual patients (i.e., inter–patient variability) occur even with the same PTV prescription. However, our results clearly demonstrated that inconsistent GTV dose is not unique to the PTV concept. Other methods that avoid the PTV concept in SBRT planning equally suffer from inconsistent GTV doses. Specifically, robust optimization that replaces the PTV concept by the worst case method also shows inconsistent GTV dose. This was evidenced by the equivalent variances of GTV *D*_*98*_*, D*_*50*_ and *D*_*2*_ among all PTV and WCS–optimized plans (Table 1). In principle, one would expect zero or minimal variability of GTV *D*_*98*_ at and close to the prescription point of GTV *D*_*98*_ or ITV *D*_*98*_ in the WCS–optimized plans. Recall that robust optimization in this study was implemented to ensure the prescription dose in the worst case scenario, that is, the GTV *D*_*98*_ was optimized to equal to or at least 54 Gy in the worst case scenario but it could be any values > 54 Gy in other scenarios. Since the nominal scenario does not necessarily coincide with the worst case scenario, and in fact hardly does, GTV *D*_*98*_ does not necessarily arrive exactly at 54 Gy in the nominal scenario and hence variability. On the other hand, any renormalization made to equalize GTV *D*_*98*_ to 54Gy in the nominal scenario would invalidate the plan robustness that was achieved to ensure the prescription dose for the worst case scenario.

When Lacornerie et al. [11] initially argued against type–B dose engine for dose optimization using the PTV concept, they claimed “the GTV will be overexposed when it moves into the regions with increased photon fluence” but without providing results to assess the magnitude of the matter. Following this line of argument, if type–B dose engine did induce excessive photon fluence in the low density PTV border one would expect the dose received by the GTV to be higher in other respiratory phases than in the planning phase. We therefore followed the phase–to–phase changes in the GTV doses. Our results show that all GTV dose parameters, except for *D*_*2*_ using the mid–ventilation concept, were statistically equal among the ten 4DCT images for the PTV–optimized plans. Guckenberger et al. [24] previously optimized for the PTV coverage *D*_*95*_ on the end–exhale CT, in which case the type–B dose engine would in principle drive the optimizer to deposit the maximal fluence at the opposite end–inhale position. Interestingly, the authors found no significant GTV dose differences when these plans were recalculated on the end–inhale CT. Maximum differences of 6.9 ± 3.1% and 2.4 ± 1.8% for GTV *D*_*99*_ and *D*_*50*_ were reported, respectively. This study observed smaller maximum differences of 2.7 ± 1.4% and 0.9 ± 0.5% for GTV *D*_*98*_ and *D*_*50*_, respectively. The discrepancy is presumably attributed to the different planning CT datasets (end–exhale vs. AIP images) for which the fluence optimization were carried out.

Here, we attempt to offer an explanation to the negligible GTV dose difference among breathing phases from the principles of conventional radiotherapy and SBRT. In conventional VMAT–based radiotherapy, a uniform dose profile (e.g., +/− 5%) across the PTV is often demanded and achieved by a fluence profile that is typically characterized with horns at the PTV edge to compensate for the beam penumbra. Thus, the GTV may experience an increase of fluence when it moves towards the PTV border. The magnitude of this fluence horn increases from water density to lung density to counterbalance the deteriorating condition of charged particle equilibrium. By contrast, SBRT allows higher dose in the tumor center (as much as 167% when normalized to the maximum dose at 60% on the PTV surface). In this case, the “horn” effect diminishes as the demand of photon fluence is counterbalanced by the allowed lower dose to the region around the PTV edge. The other possible reason could be that commercial planning system generally switches the type–A dose engine to type–B dose engines only at certain steps for fluence correction during the dose optimization and in final dose calculation.

Additionally, we examined the variances of different GTV dose parameters among the ten respiratory phases. Our hypothesis is that if type–B dose engine did drive up the photon fluence in the PTV–optimized plans the inter–phase variability of these GTV dose parameters would become significantly different. This hypothesis is based on the fact that individual patients have different characteristics (e.g., tumor size, motion amplitude, lung density, etc) and hence the extent to which the photon fluence were to be driven up would vary substantially. When the GTV moves in different spatio–temporal positions of the respiratory cycle it would receive photon fluence of varying degree from phase to phase that is patient dependent. Nonetheless, we found that both PTV and WCS optimizations resulted in equal variances of all GTV dose parameters among the ten respiratory phases. Interestingly, the inter–quartile ranges (IQR) of GTV *D*_*98*_ resulting from WCS optimized plans using all 4DCT images were found to be more variable than from other PTV–optimized plans. This large but insignificant variability of GTV *D*_*98*_ is hypothesized to have originated from the specific worst case optimization method. Compared to the voxel–wise and objective–wise robust methods, the composite worst case method implemented by the RayStation planning systems behaves to maximally minimize the objective value on the worst case scenario at the cost of higher objective values and thus larger dosimetric fluctuation in many other possible scenarios [25]. Since the worst case scenario may correspond to different breathing phases with different patient characteristics, relatively large variability of *D*_*98*_ among breathing phases was observed. Nonetheless, by WCS optimization, particularly using all 4DCT images, the highest robustness was achieved to prevent the dose limits in the normal tissues from being exceeded when the target is displaced into different respiratory positions.

As the final validation, we compared the optimized dose on a single CT and the recalculated doses summed over all 4DCT images. Such comparisons offer clarifications to two important issues concerning the non–consistency of PTV concept in lung SBRT. Firstly, if type–B dose engine induced excessive fluence in PTV–based optimization, the GTV would eventually accumulate significant higher dose when it moved into different breathing phases. However, no clear indication of overexposure to the GTV can be associated with PTV–based optimization (Table 3). The GTV *D*_*50*_ and *D*_*2*_ obtained from PTV–optimized plans for the ITV and mid–ventilation concepts changed by 0.3 Gy only after dose summation and on the contrary decreased rather than increased. The significant increase of GTV *D*_*98*_ in the PTV–optimized plans based on the ITV concept does not appear to be related to the type–B dose engine because it did not occur to the other PTV–optimized plans that adopted the mid–ventilation concept. Instead, it was presumably caused the systematic change in using the AIP images for dose optimization to the mid–ventilation images for dose accumulation. For the rather extreme situation using the end–exhale CT for fluence optimization, neither did Guckenberger et al. [24] observe serious problem of excessive build up of photon fluence at the opposite end–inhalation that caused a significant change in the overall GTV dose either. More interestingly, the authors too found an increase rather than a decrease in the summed GTV dose (presumably *D*_*95*_) by less than 1% or 0.7 Gy only. Among all GTV dose parameters, *D*_*50*_ appears to be the most robust against changes showing no statistical significance except for the ITV–based robust optimization. Based on these results, we conclude that type–B dose engine, per se, does not significantly increase the GTV dose. The significantly higher GTV dose in the PTV–optimized plans than WCS optimized plans is rather a direct consequence of the prescription method.

Secondly, equal variances of the GTV dose parameters among the PTV and WCS–optimized plans are still observed after dose summation over the ten 4DCT images. The inter–patient variability (one standard deviation) changes only by 0.1 Gy after dose summation in all but the GTV *D*_*98*_ of the WCS–optimized plans (0.9 Gy). This simply means that the inconsistency of GTV dose cannot be easily resolved by migrating from the PTV concept to robust optimization irrespective of the type–B dose engine [1, 14]. For the same reasoning, we would argue that using two classes of dose engines, a type–A for fluence optimization followed by a type–B for subsequent dose calculation and renormalization will not resolve the inconsistent GTV dose either. We would further argue that PTV concept, in its very design to account for geometric uncertainty, shall not be considered as a pitfall. Consistency of clinical outcome report shall not be compromised provided that the advanced dose engines are used to estimate and report the GTV dose parameters following the ESTRO ACROP recommended guidelines [11].

### PTV– and WCS–based SBRT by GTV median dose renormalization

Lebredonchel et al [14] suggested that prescribing based on 50% mass of the PTV can somewhat stabilize variability of the target dose but they concluded further that moving away from the PTV concept for prescription remains the only solution if using type–B dose engine. They came to this conclusion because the GTV median dose *D*_*50*_ differs substantially with variable lung density and tumor size when prescription is done to the PTV. However, this conclusion is considered as partly true only because our results already showed that other PTV–free concept by the worst case method does not stabilize the target doses either when the ICRU recommended prescription by coverage (i.e., GTV *D*_*98*_ or ITV *D*_*98*_) was followed. Instead, the prescription method has the major impact on the variability of GTV dose. After renormalization based on GTV *D*_*50*_, the separations of the DVH families became much packed together for all plans optimized using different concepts (Fig. 4), as compared to those obtained from prescription by coverage (Fig. 1). The resulting SDs of *D*_*98*_ and *D*_*50*_ and *D*_*2*_ are limited to 1 Gy for PTV– and 1.4 Gy for WCS–optimized plans, respectively. Focusing on the concept of ITV as motion encompassing, Lang et al. similarly showed that the SDs of PTV *D*_*98*_ and *D*_*50*_ and ITV *D*_*98*_ of 38 patients are limited to 1.5 Gy after ITV *D*_*50*_ renormalization to 57 Gy [18]. They also showed that the ITV *D*_*50*_ renormalization is superior to renormalization by ITV/PTV coverage *D*_*98*_ as it can reduce the variability of PTV and ITV dose parameters among delivery techniques (dynamic conformal arc vs. VMAT). More importantly, the differences of GTV *D*_*98*_ and *D*_*50*_ and *D*_*2*_ among optimized plans based on the PTV concept and the WCS method (Table 4) were found to reduce markedly. These results are still valid despite the variation of tumor position in the respiration cycle, with GTV *D*_*50*_ being the only dose parameter that showed statistically significant difference. However, the absolute difference of 0.2 Gy is deemed clinically unimportant. Same as the results of coverage prescription, the median dose turned out to be the most robust against uncertainty of tumor position among other GTV dose parameters.

The effect of GTV *D*_*50*_ renormalization is also marked at the phase to phase level (Fig. 5). The median of all GTV dose parameters became much closer among the plans that adopted different concepts for setup and motion compensation. Compared to the prescription by coverage method recommended by ICRU 91 report, the maximum inter–phase difference of GTV *D*_*98*_ was reduced by 2.4, 4.8 and 2.4% and 1.0% for PTV optimization by the ITV and mid–ventilation concepts, and WCS optimization to the ITV and GTV, respectively.

In summary, when SBRT plans are directly prescribed or renormalized to the GTV median dose *D*_*50*_

1. the consistency of GTV dose across the near–minimum, median, and near–maximum points is significantly improved, i.e. reduced inter–patient variability
2. harmonization of GTV dose is made possible for lung SBRT plans that adopt different concepts to handle geometric uncertainty caused by respiratory motion.

The first point simply implies that one can continue with the PTV concept for dose planning. The second point implies that consistent GTV dose shall be ensured between SBRT centers employing either the PTV concept or the worst case scenario concept in dose planning, and different delivery techniques as indicated by Lang et al. [18].

On the other hand, one may question the value of robust optimization concerning its computational overheads, if by *D*_*50*_ GTV prescription alone can simply harmonize the GTV dose among optimization solutions. From the normal tissue dose perspective, our phase–by–phase analysis indicates that WCS optimization in general improved the dosimetric robustness, resulting in the fewest number of dose deviations from the OAR limits. Furthermore, lower NL_V5_ and MLD (Table 3) during respiration were constantly observed in the WCS optimization group regardless of the prescription method. In particular, WCS optimization to the GTV using all 4DCT images resulted in the lowest normal tissue dose and highest robustness against deviation of normal tissue dose limit among all optimization methods.

### Limitation of the study

This study was designed by assuming the same amount of geometric uncertainties from tumor motion and patient setup in the calculation of the PTV and in the definition of the WCS parameters. Nonetheless, our results considered exclusively the uncertainty of tumor position due to breathing motion. The validity of our results shall hold because uncertainty of respiratory motion, which is considered as systematic in our phase–to–phase analysis for the GTV dose changes, is much greater than that of setup limited to millimeter accuracy with stereotactic image guidance.

The other limitation is the small number of patients which may subject our results to bias. Only 2 out of 13 patients showed tumor motion more than 1 cm. It is unclear whether our dosimetric results will remain unchanged if more patients with larger amplitude of tumor motion are included.

We also acknowledge that the exact formulation of the robustness optimization may have an influence on the dosimetric results [22]. Despite the numerous robustness optimization algorithms, there is only one commercial planning system that makes robust optimization available for clinical use. This study, like many other previous ones, was based on the worst case scenario optimization from the same planning system. Lastly, this study focused on a certain type (convolution–superposition) and class (type–B) of dose engine. Systematic difference between Monte Carlo and type B dose engines is well known especially in cases where extreme electron charged disequilibrium exists [26]. Further investigation with Monte Carlo dose engine is warranted to generalize the present findings.

## Conclusions

The pitfalls of PTV concept have no association with type–B dose engine in lung SBRT. Inconsistent target dose is not unique to the PTV concept but the worst case method implemented in the robust optimization. Prescription by coverage, regardless to the PTV *D*_*95*_ or GTV *D*_*98*_ in common practice has the major impact on the consistency of GTV dose. GTV median dose prescription or renormalization can effectively decrease the inter–patient and inter–optimization method (PTV and worst case scenario) variability of GTV dose.

## Data Availability

The datasets related to patient information are not available. Other data used and / or analyzed during the present study are available from the corresponding author on reasonable request.

## Abbreviations

SBRT: Stereotactic body radiotherapy
4DCT: Four dimensional computed tomography
AIP: Averaged intensity projection
MidV: Mid-ventilation
VH: van Herk’s margin recipe
RO: Robust optimization
WCS: Worst case scenario
GTV: Gross tumor volume
ITV: Internal target volume
PTV: Planning target volume
PTV_ITV_: SBRT plan by PTV + ITV concept using AIP-CT
PTV_VH_: SBRT plan by PTV + VH concept using MidV-CT
WCS_ITV_: SBRT plan by WCS + ITV concept using AIP-CT
WCS_GTV_: SBRT plan by WCS + GTV concept using 4DCT
